# Effects of menstrual disorders and dysmenorrhea on cardiovascular disease: a Mendelian randomization study

**DOI:** 10.3389/fendo.2024.1302312

**Published:** 2024-02-05

**Authors:** Sijia Lai, Qiubai Jin, Dayang Wang, Tianli Li, Xian Wang

**Affiliations:** ^1^ Institute of Cardiovascular Disease, Dongzhimen Hospital, Beijing University of Chinese Medicine, Beijing, China; ^2^ Guang’anmen Hospital, China Academy of Chinese Medical Sciences, Beijing, China; ^3^ National Integrated Traditional and Western Medicine Center for Cardiovascular Disease, China-Japan Friendship Hospital, Beijing, China

**Keywords:** menstrual disorders, dysmenorrhea, cardiovascular disease, Mendelian randomization, FinnGen

## Abstract

**Background:**

Observational studies have demonstrated associations between menstrual disorders, dysmenorrhea, and cardiovascular disease (CVD). However, it remains unclear whether these associations are causal. This study is to investigate whether menstrual disorders and dysmenorrhea causally affect the risk of CVD.

**Methods:**

The summary data for menstrual disorders (excessive menstruation and irregular menses) and dysmenorrhea were obtained from FinnGen study, summary data for CVD were obtained from UK Biobank and meta-analysis. The inverse-variance-weighted method was mainly used in the Mendelian randomization for causality analysis. Sensitivity analyses were performed by several methods under different model assumptions.

**Results:**

Genetic liability to excessive menstruation was associated with higher risk of atrial fibrillation (odds ratio (OR), 1.078 [95% confidence interval (CI), 1.015-1.145]; *P*=0.014), but a lower risk of hypertension (OR, 0.994 [95% CI: 0.989-0.999]; *P*=0.016). Irregular menses was associated with higher risk of atrial fibrillation (OR, 1.095 [95% CI: 1.015-1.182]; *P*=0.02), hypertension (OR, 1.007 [95% CI: 1.000-1.013]; *P*=0.047), myocardial infarction (OR, 1.172 [95% CI: 1.060-1.295]; *P*=0.02), ischemic heart disease, (OR, 1.005 [95% CI: 1.000-1.010]; *P*=0.037) and coronary heart disease (OR, 1.004 [95% CI: 1.001-1.008]; *P*=0.026). Dysmenorrhea was associated with higher risk of atrial fibrillation (OR, 1.052 [95% CI: 1.014-1.092]; *P*=0.008) and Ischemic stroke (cardioembolic) (OR, 1.122 [95% CI: 1.002-1.257]; *P*=0.046). After Benjamini-Hochberg correction, irregular menses was associated with higher risk of myocardial infarction.

**Conclusion:**

We confirmed a causal relationship of excessive menstruation, irregular menses and dysmenorrhea on cardiovascular outcomes independent of sex hormone levels, with an emphasis on the link between irregular menses and myocardial infarction. These clinical features can be utilized as markers to identify women at higher risk of developing CVD in the future, recommending early clinical intervention of menstrual diseases.

## 1 Background

Cardiovascular disease (CVD) is the leading cause of mortality for women worldwide and accounted for 35% of total deaths in women in 2019 (1, 2). The United Nations General Assembly Sustainable Development Goal target 3.4 is to reduce premature mortality (including CVD and cancer) from non-communicable diseases by a third by 2030 (3). However, the global research on female CVD is still insufficient (1).

Regular menstruation reflects the normal function of hypothalamus-pituitary-ovary axis, which is an important sign of women’s overall health (4). Growing evidence shows that women with self-report or physician report of irregular menstrual cycle (cycle length <21 days; 36 days or longer; or physician-coded oligomenorrhea, anovulatory cycles, or irregular menses) have an increased incident or mortality of CVD (5–7), and early female reproductive characteristics can serve as markers for CVD risk (8). Nevertheless, evidence linking menstrual characteristics throughout the entire reproductive period to CVD is still limited, and the causal effect is still unclear. Several cohort studies have found that irregular, long (≥35 days) or short (≤21 days) menstrual cycle length is associated with higher risk of CVD later in life, with only a small proportion driven by hypercholesterolemia, chronic hypertension, and type 2 diabetes (8, 9). For example, those with greater menstrual cycle irregularity (ie, “usually or always irregular” or no periods) and longer menstrual cycle length (40 or more days or too irregular to estimate) in both early adulthood and mid-adulthood have higher rate of CVD (8). Specifically, the analysis of 80,630 women on Nurses’ Health Study II (NHS II) found that those who reported usually irregular or always irregular/no period at 29-46 ages had an 27% and 54% increased risk for CHD after 24 years of follow-up, respectively (8). Similar to the previous NHS study, 25% and 67% higher risk was observed among those who reported usually irregular or very irregular cycles at 20-35 ages (7). A retrospective matched cohort study of 704,743 women from the UK found that irregular menstrual cycle relates to increased risk of ischemic heart disease (IHD) and heart failure, and frequent menstrual cycle relates to increased risk of complex CVD and cerebrovascular disease (10). Primary dysmenorrhea often debilitating that affects 45%~95% of menstruating women (11), while evidence showed an increased risk of CVD in chronic pain (12). Given that menstrual disorders and dysmenorrhea are common clinical manifestation of various diseases (11, 13–15) and are influenced by hormone levels, endocrine (16), and metabolic factors (17), the role of these confounding factors cannot be completely ruled out in observational studies, and the definite causality between menstrual disorders, dysmenorrhea and CVD is still unclear. Therefore, data and high-level evidence from large sample studies are still needed to fill this gap. However, the randomized controlled trial for causal inference is impractical to carry out in this situation.

Two sample Mendelian randomization (TSMR) mimics randomization based on the random distribution of genetic variants during gametogenesis, offering an alternative to randomized controlled trials for investigating causal relationships between exposures and outcomes (18). In this study, we used the latest available genome-wide association studies (GWAS) database published in 2023 for TSMR analysis to investigate the possible causality between menstrual disorders, dysmenorrhea, and CVD from a genetic perspective, which is of great public health significance for the prevention of CVD in women.

## 2 Methods

### 2.1 Study design

**Figure 1:**
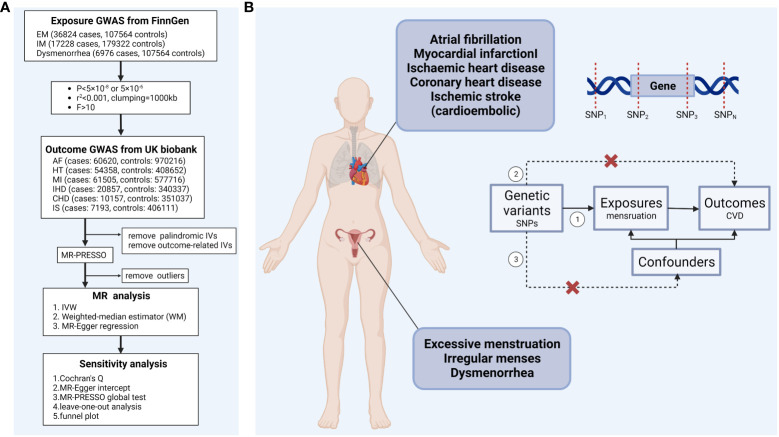
**(A)** Schematic overview of the study design **(B)** Mendelian randomization analysis assumptions ①Relevance assumption. ②Exclusion restriction. ③Independence assumption. IVW, inverse-variance-weighted; EM, excessive menstruation; IM, irregular menses; AF, atrial fibrillation; HT, hypertension; MI, myocardial infarction; IHD, ischemic heart disease; CHD, coronary heart disease; IS, ischemic stroke (cardioembolic).

TSMR was used to analyze the causal relationship between menstruation disorders (excessive menstruation (EM), irregular menses (IM)), dysmenorrhea and CVD (atrial fibrillation (AF), hypertension (HT), myocardial infarction (MI), IHD, coronary heart disease (CHD), and ischemic stroke (cardioembolic) (IS)). A schematic overall design of this study is shown in **Figure 1A** (created with BioRender.com.). To obtain reliable results, three hypotheses need to be satisfied when performing TSMR analysis (1): genetic variants strongly correlate to exposure factors (2); genetic variants are not correlate to confounders; and (3) genetic variants affect the outcome exclusively through the exposure factors, that is, horizontal pleiotropy is not allowed (**Figure 1B**). In this study, confounders including smoking, low density lipoprotein, diabetes, arrhythmias, blood pressure, atherosclerosis, sex hormones levels, and ever used hormone-replacement therapy. Genetic variants that satisfy these three hypotheses can be included in TSMR analysis as instrumental variables. This Mendelian randomization (MR) study was performed following the STROBE-­MR (Strengthening the Reporting of Observational Studies in Epidemiology using Mendelian Randomisation) guideline (19).

### 2.2 Data sources for menstruation

This study used publicly available, summary-level genome-wide association study (GWAS) data. The GWAS data set for menstruation was obtained from FinnGen study (https://r9.finngen.fi/) released in May 2023 (20). The ongoing FinnGen study initiated in 2017, combines genetic data from the Finnish biobanks with health record data from Finnish health registries (21). The diagnostic criteria of excessive menstruation, irregular menses, and dysmenorrhea were based on the International Classification of Diseases (ICD) standards. Specifically, “excessive menstruation” was defined as “ICD-10-N92: Excessive, frequent and irregular menstruation”; “irregular menses” was defined as “ICD-10-N91: Absent, scandy and rare menstruation; ICD-10-N92.1: Excessive and frequent menstruation with irregular cycle; ICD-10-N92.5: Other specified irregular menstruation; ICD-10-N92.6: Irregular menstruation, unspecified”; and “dysmenorrhea” was defined as “ICD-10-N94: Pain and other conditions associated with female genital organs and menstrual cycle” involving primary dysmenorrhea, secondary to endometriosis, mittelschmerz, and other conditions.

The GWAS statistics involve 36,824 cases and 107,564 controls of excessive menstruation, 17,228 cases and 179,322 controls of irregular menses, 6,976 cases and 107,564 controls of dysmenorrhea.

### 2.3 Data source for CVD

When we identified multiple GWAS for a single trait, only the latest and the largest study with replication was used. The summary statistics for AF were obtained from a meta-analysis of the GWAS of European-ancestry participants (60,620 cases and 970,216 controls) from 6 studies (22) (The Nord-Trøndelag Health Study (HUNT), deCODE, the Michigan Genomics Initiative (MGI), DiscovEHR, UK Biobank, and the AFGen Consortium)). The summary statistics for MI were obtained from a meta-analysis of the GWAS of European-ancestry participants (61,505 cases and 577,716 controls) from 2 cohort studies (23) (CARDIoGRAM+C4D Consortium and the UK BioBank). The summary statistics for cardioembolic stroke were obtained from a meta-analysis of the GWAS of European-ancestry participants (7,193 cases; 406,111 controls) from 17 studies (24) (CHARGE, METASTROKE, SIGN, DECODE, EPIC-CVD, Young Lacunar DNA, SIFAP, INTERSTROKE EUR, HVH1, Glasgow, CADISP, Barcelona, FINLAND, SAHLSIS, MDC, HVH2, ICH). Among them, less than 187 individuals were overlap between the exposure and cardioembolic stroke data. The heterogeneity *P* values of the meta-analyses were not significant. Genetic association estimates for hypertension were obtained from the UK Biobank, based on the UKB GWAS pipeline set up for the Medical Research Council Integrated Epidemiology Unit (MRC IEU) (25) (https://gwas.mrcieu.ac.uk/). (54,358 cases and 408,652 controls); IHD (20,857 cases and 340,337 controls), CHD (10,157 cases and 351,037 controls) from the IEU GWAS pipeline using UKB data. To minimize ancestry mismatches, we restricted the analytical cohort to individuals of European descent. Further details on data sources are included in **Table 1**.

**Table 1:** Summary of the GWAS included in this two-sample MR study.

Phenotype	ID	Sample size	Number of SNPs	Consortium/Study	Ancestry	Sex	Year
Exposure data							
Excessive menstruation	finngen_R9_N14_ MESNRUIRREG	36,824 cases 107,564 controls	20,150,156	FinnGen	European	Females	2023
Irregular menses	finngen_R9_N14_ MENSIRREG	17,228 cases 179,322 controls	20,160,830	FinnGen	European	Females	2023
Dysmenorrhea	finngen_R9_N14_ FEMGENPAIN	6,976 cases 107,564 controls	20,136,432	FinnGen	European	Females	2023
Outcome data							
Atrial fibrillation	ebi-a-GCST006414	60,620 cases 970,216 controls	33,519,037	Meta-analysis	European	Males and Females	2018
Hypertension	ukb-b-12493	54,358 cases 408,652 controls	9,851,867	MRC-IEU	European	Males and Females	2018
Myocardial infarction	ebi-a-GCST011364	14,825 cases 2,680 controls	10,290,368	Meta-analysis	European	Males and Females	2021
Ischaemic heart disease	ukb-d-I9_IHD	20,857 cases 340,337 controls	13,586,589	Neale lab analysis of UK Biobank	European	Males and Females	2018
Coronary heart disease	ukb-d-I9_CHD	10,157 cases 351,037 controls	13,295,130	Neale lab analysis of UK Biobank	European	Males and Females	2018
Ischemic stroke	ebi-a-GCST006910	7,193 cases 406,111 controls	8,271,294	Meta-analysis	European	Males and Females	2018

### 2.4 Instrument selection of exposure

To obtain moderate instrumental variables (IVs), we screened single-nucleotide polymorphisms (SNPs) of EM at genome-wide significance *P* < 5×10^–8^, IM and dysmenorrhea at genome-wide significance *P* < 5×10^–6^ as instruments and clumped these SNPs with linkage disequilibrium r^2^ < 0.001 at a 1,000 kb window to ensure no linkage disequilibrium among the included IVs. To avoid the influence of weak instrument bias on causal inference, we calculated the strength of each IVs using the formula $F=R^{2}\left(N-2\right)/\left(1-R^{2}\right)$ , and deleted SNPs with *F* < 10 to fulfill the first assumption of MR (26, 27). R^2^ is the variance of the risk of EM, IM, and dysmenorrhea explained by each instrument and N is the GWAS sample size of exposures (28). Given the more than 10 genome-wide significant SNP instruments, R^2^ was calculated as (27): $\frac{2\times EAF\times \left(1-EAF\right)\times beta^{2}}{\left(2\times EAF\times \left(1-EAF\right)\times beta^{2}\right)+\left(2\times EAF\times \left(1-EAF\right)\times N\times SE_{beta}^{2}\right)}$ , where EAF is the effect allele frequency, beta is the estimated genetic effect on exposures, SE_beta_ is the standard error of the genetic effect (29). The details of the genetic IVs are presented in **Table S1**.

To avoid horizontal pleiotropy, we excluded IVs associated with risk factors for CVD using PhenoScanner V2. Palindromic and incompatible SNPs were excluded when harmonizing the statistics of exposure and outcome. MR Pleiotropy RESidual Sum and Outlier (MR-PRESSO) test was applied to remove the outlier SNPs before each MR analysis and sensitivity analysis (30).

### 2.5 Statistical analysis

The causal association was examined using the random-effect IVW regression model, which assumes the absence of directional and horizontal pleiotropy (31). Weighted median estimator (WM) and MR-Egger regression were also used to improve the IVW estimates in a broader set of scenarios. WM assumes that less than 50% of IVs have horizontal pleiotropy (32). MR-Egger regression assumes more than 50% of IVs are affected by horizontal pleiotropy but being independent of the variant-exposure association (33). Although these two methods result in wider confidence intervals, more robust estimates can be provided.

To ensure the reliability of the results, we performed sensitivity analysis of heterogeneity and pleiotropy, using Cochran’s Q, MR-Egger intercept, and MR-PRESSO tests. Cochran’s Q-test was utilized to measure the degree of heterogeneity among genetic IVs (34). The MR-Egger intercept test was used to estimate average pleiotropic effect across the genetic IVs. If the value was non-zero, it indicated that the IVW estimate was biased (33). MR-PRSSO test reduced the effect of horizontal pleiotropy on causal inference from several perspectives: MR-PRESSO global test was applied to detect the existence of horizontal pleiotropy, MR-PRESSO outlier test was applied to correct horizontal pleiotropy via outlier removal, and MR-PRESSO distortion test was applied to test whether there was a significant difference between the causal estimates before and after outlier correction (30). The distribution was set to 1000, and the statistically significant *P*-value of sensitivity analysis was set as 0.05.

In addition, we conducted a leave-one-out analysis of the significant estimates to check for any bias in the causal relationship of the TSMR analysis caused by a single SNP, and assessed the probable directional pleiotropy by performing a funnel plot of the significant estimates. TSMR analyses were performed with R version 4.2.2 using the ‘TwoSampleMR’ package version 0.5.7.

To correct for multiple testing, we used Benjamini-Hochberg method, which applies a false discovery rate (FDR) (35). When TSMR analysis satisfy FDR-adjusted statistic significant (*P* < 0.05) or nominal *P* < 0.05 by two or more TSMR methods, the result is considered robust. Meanwhile *P* < 0.05 was regarded as nominally significant, suggesting potential causality between menstruation and CVD (36).

## 3 Results

### 3.1 Selection of instrumental variables

Complete results were presented in **Supplementary Tables**. In total, 19 SNPs, 24 SNPs and 25 SNPs were selected to genetically predict EM, IM and dysmenorrhea, respectively. The *F*-statistics of all SNPs were greater than 10, indicating that there is no bias caused by weak IVs (**Table S1**). SNPs related to diseases and traits were recorded at the significance level (*P* < 1 × 10^−5^) in PhenoScanner. The rs17679286, rs3197999 and rs66531120 were associated with “coronary artery disease”, “Diastolic blood pressure, and coronary artery disease”, “Vascular or heart problems diagnosed by doctor: high blood pressure”, respectively. The rs11031005 was associated with “Sex hormone levels, and ever used hormone-replacement therapy”. These four SNPs were removed before TSMR analysis (**Table S2**). Potentially pleiotropic SNPs were also excluded using MR-PRESSO outlier test. In specific, rs9964201, rs144737447, rs2458, and rs10496768 was excluded from the analysis of EM-HT, IM-AF, IM-MI, and dysmenorrhea-HT, respectively (**Table S2**). Moreover, SNP rs1806176 overlapped in EM and IM. There was no overlap between EM and dysmenorrhea, nor IM and dysmenorrhea.

### 3.2 Causal effect in the main analysis

**Figure 2:**
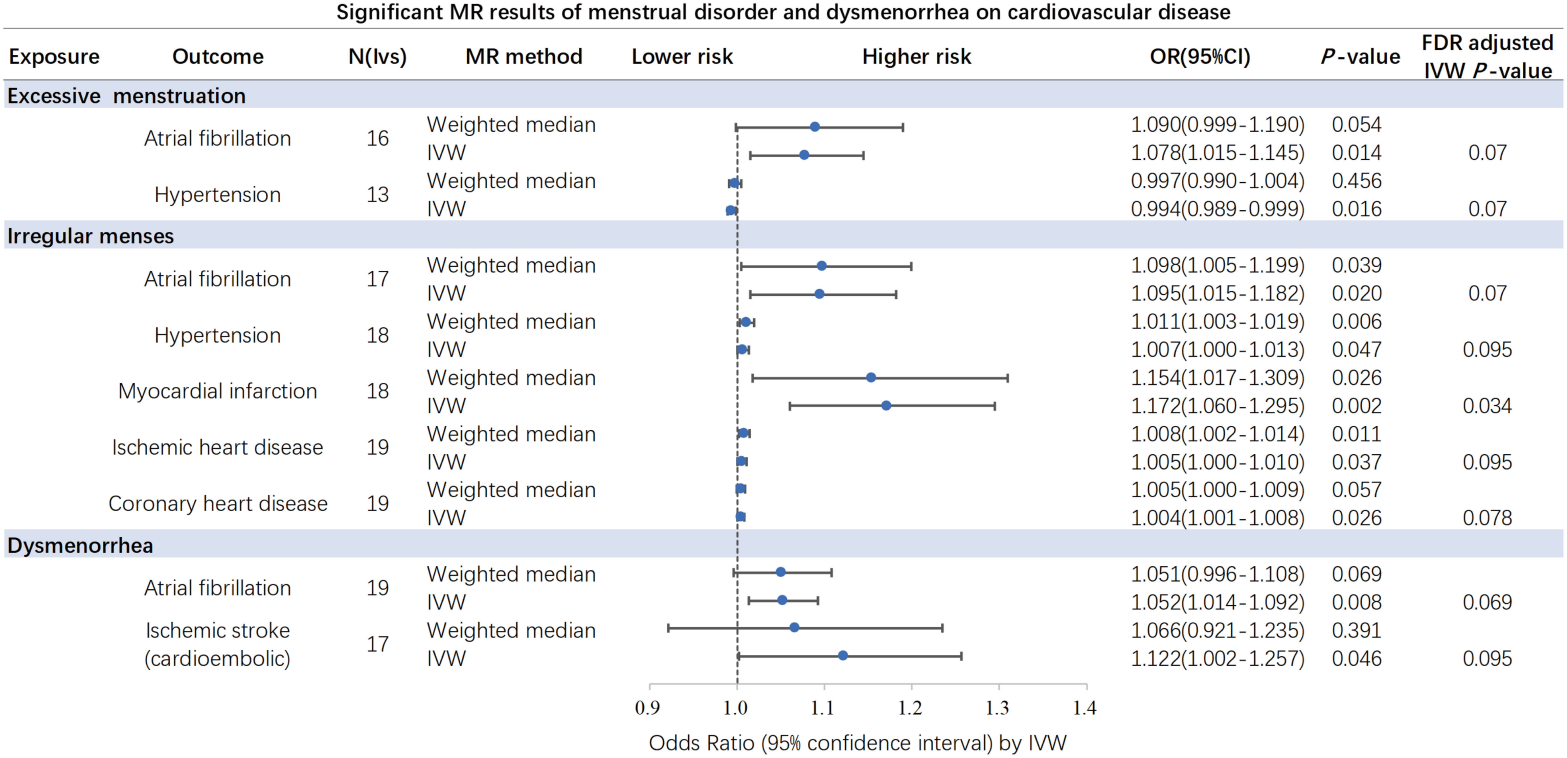
Significant and nominal significant causal associations of excessive menstruation, irregular menses and dysmenorrhea on cardiovascular disease. After Benjamini-Hochberg correction, the IVW method indicated that irregular menses was associated with higher risk of myocardial infarction. IVW, inverse-variance-weighted. .

**Figure 3:**
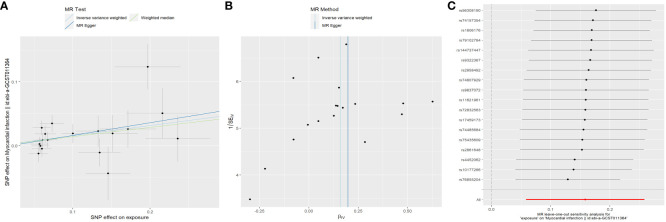
Plots of Mendelian randomization estimates showing the causal effects of genetically predicted menstrual disorder and dysmenorrhea on cardiovascular disease. **(A)** Scatter plot from genetically predicted IM on MI; **(B)** Funnel plot from genetically predicted IM on MI; **(C)** Leave-one-out plot from genetically predicted IM on MI. IM, irregular menses; MI, myocardial infarction.

Overall, the results of the MR methods were generally consistent (**Table S3**). Nine potential causal associations between menstrual disease and CVD were preliminary identified by the IVW and WM. The scatter plots of nominal significant estimates from genetically predicted EM, IM and dysmenorrhea on CVD are included in **Supplementary Figures S1**-**S3**. Specifically, the TSMR analysis showed that genetically determined EM was associated with higher risks of AF, with odds ratio (OR): 1.078 (95% confidence interval [CI]: 1.015-1.145; *P*=0.014), but a lower risk of hypertension (OR: 0.994; 95% CI: 0.989-0.999; *P*=0.016). IM was associated with higher risks of AF (OR: 1.095; 95% CI: 1.015-1.182; *P*=0.02), hypertension (OR: 1.007; 95% CI: 1.000-1.013; *P*=0.047), MI (OR: 1.172; 95% CI: 1.060-1.295; *P*=0.02), IHD (OR: 1.005; 95% CI: 1.000-1.010; *P*=0.037) and CHD (OR: 1.004; 95% CI: 1.001-1.008; *P*=0.026). Dysmenorrhea was associated with higher risks of AF (OR: 1.052; 95% CI: 1.014-1.092; *P*=0.008) and IS (OR: 1.122; 95% CI: 1.002-1.257; *P*=0.046). After Benjamini-Hochberg correction, the IVW method indicated that IM was associated with higher risk of MI (*P*=0.034) (**Figure 2**). The scatter plot, funnel plot and leave-one-out plot from genetically predicted IM on MI are shown in **Figure 3**.

### 3.3 Causal effect in the sensitivity analyses

#### 3.3.1 Excessive menstruation

Cochrane’s Q test did not suggest evidence of heterogeneity in AF (*P* = 0.540) and HT (*P* = 0.414), nor did MR-Egger intercept test observe horizontal pleiotropy in AF (*P* = 0.678) and HT (*P* = 0.253). Similarly, no evidence of horizontal pleiotropy was detected in the MR-PRESSO global test in AF (*P* = 0.578) and HT (*P* = 0.402).

#### 3.3.2 Irregular menses

Cochrane’s Q test suggest no evidence of heterogeneity in HT (*P* = 0.061), MI (*P* = 0.145), IHD (*P* = 0.092) and CHD (*P* = 0.057), but showed heterogeneity in AF (*P* = 0.044). As we used the random-effects IVW as main result, heterogeneity is acceptable (37). MR-Egger intercept test did not observe horizontal pleiotropy in AF (*P* = 0.903), HT (*P* = 0.447), MI (*P* = 0.726), IHD (*P* = 0.893) and CHD (*P* = 0.450). Similarly, no evidence of horizontal pleiotropy was detected in the MR-PRESSO global test of AF (*P* = 0.072), HT (*P* = 0.061), MI (*P* = 0.161), IHD (*P* = 0.106) and CHD (*P* = 0.065).

#### 3.3.3 Dysmenorrhea

Cochrane’s Q test did not suggest evidence of heterogeneity in AF (*P* = 0.489) and IS (*P* = 0.996), nor did MR-Egger intercept test observe horizontal pleiotropy in AF (*P* = 0.346) and IS (*P* = 0.531). Similarly, no evidence of horizontal pleiotropy was detected in the MR-PRESSO global test in AF (*P* = 0.527) and IS (*P* = 0.995).

Taken collectively, the Cochran Q test for most significant traits did not demonstrate heterogeneity (**Table 2**). Although outliers were detected, no significant difference in the estimate was found before and after the removal of outliers (MR-PRESSO distortion test *P* > 0.05). Besides, the MR Egger intercept of all traits indicated no horizontal pleiotropy, thereby confirming the causal inference of the MR estimates (**Table S4**). In addition, we found that excluding one SNP at a time did not materially alter the risk estimates of genetically predicted EM, IM and dysmenorrhea on the risk of CVD, indicating that potential driving SNPs are unlikely to bias the causal association. The leave-one-out analysis, together with funnel plot, further validated the robustness of our findings (**Supplementary Figures S4**-**S9**).

**Table 2:** Results of heterogeneity and sensitivity test of significant and nominal significant causal associations.

Exposure	Outcome	MR methods	*P* of pleiotropy	*P*-value of Cochran Q
Excessive menstruation	Atrial fibrillation	MR Egger	0.678	0.478
		IVW		0.540
	Hypertension	MR Egger	0.253	0.447
		IVW		0.414
Irregular menses	Atrial fibrillation	MR Egger	0.903	0.031
		IVW		0.044
	Hypertension	MR Egger	0.447	0.057
		IVW		0.061
	Myocardial infarction	MR Egger	0.726	0.115
		IVW		0.145
	Ischaemic heart disease	MR Egger	0.893	0.069
		IVW		0.092
	Coronary heart disease	MR Egger	0.450	0.053
		IVW		0.057
Dysmenorrhea	Atrial fibrillation	MR Egger	0.346	0.484
		IVW		0.489
	Ischemic stroke (cardioembolic)	MR Egger	0.531	0.995
		IVW		0.996

## 4 Discussion

This is the first attempt to investigate the causal relationship between EM, IM, dysmenorrhea and CVD risk through a TSMR analysis using GWAS summary-level statistics. Previous studies found that women with irregular menstrual cycle have an increased incident or mortality of CVD. In alignment with earlier assessments based on NHS, our TSMR analyses found evidence of causal relationship —that EM, IM and dysmenorrhea are likely to be causal determinants of CVD risk, especially for irregular menses and myocardial infarction.

TSMR uses genetic variants as IVs of exposure, providing an unconfounded and minimized reverse causality test between the particular phenotype and disease (18, 21, 38). In our study, we strictly screened the IVs and selected robust genetic instruments with *F* statistics that were larger than the normally used value of 10. Since the MR Egger intercept of all traits indicated no horizontal pleiotropy, using IVW as the primary method for analyzing causality is appropriate (31), and the consistency of our results across multiple MR methods adapts to various assumptions about genetic pleiotropy and strengthens the causal inference of our analysis (39, 40). To reduce false discovery of the findings, we used Benjamini-Hochberg correction, which gave us only one estimate with a significant adjusted p-value. However, other potentially important clinical estimates with IVW-derived *P* value < 0.05 should also be taken seriously (40). Given the relatively small sample size of the dysmenorrhea data, related analyses should be replicated when larger datasets are available (41).

Observational studies have shown associations between menstrual disorders and CVD. Menstrual cycle characteristic self-report has been validated in other studies and is regarded as reliable (6). An irregular menstrual cycle has been observed to be associated with an increased risk of hypertension (OR: 1.07, 95%CI: 1.03-1.11) (10), aligning with our MR findings (OR: 1.007; 95% CI: 1.000-1.013). However, frequent menstrual cycles have been linked to heightened hypertension risks (10), a finding that contradicts our MR results. The increased risk of CVD in postmenopausal women is generally believed to be related to a lack of protection from sex hormones (42). A mendelian randomization found that sex hormone-binding globulin, testosterone and oestradiol may causally affect risk of hypertension and coronary atherosclerotic outcomes (43). Therefore, the sex hormone level, hormone-replacement therapy, and hormonal contraceptives were regarded as potential confounders. In the analysis of EM and hypertension, we excluded SNPs related to sex hormone, hence the removal of confounding sex hormones may contribute to the contrasting result. Our MR analysis showed that EM, and IM are associated with a higher risk of AF. Similar results were observed in a perspective study of 58,056 women from UK Biobank that during the median 11.8 years of follow-up, each of irregular menstrual cycle (cycle length ≤21 days or ≥35 days), long (≥35 days) or short (≤21 days) cycle length is associated with AF risk (9). Moreover, irregular and long menstrual cycles (≥40 days or too irregular to estimate) are associated with higher risk of premature mortality, with strongest relations for deaths related to CVD among cancer, CVD, and other causes (6). A recent study reported increased rates of coronary heart disease and MI observed among women who experienced short cycle length (9). This is similar to our MR analysis that IM increased the risk of IHD, CHD and MI. In addition, observational study shows that stroke does not play a significant role in the relationship between menstrual cycle disorder and CVD (7). Our TSMR analysis did not support a causal relationship between EM, IM and IS either, which is in accordance with prior literature, that is, short cycle length (9) and irregular menstrual cycle (10) is no significant relation to stroke. Intriguingly, we found that dysmenorrhea is causally associated with an increased risk of IS (cardioembolic), possibly driven by increased risk of AF. The underlying mechanism between menstrual disorders and CVD is multi-factorial and interrelated. Menstrual disorders may increase the risk of CVD through mechanisms affecting metabolism and hormone levels, such as insulin resistance, hormonal imbalance, and endothelial dysfunction. For instance, increased insulin levels can stimulate the production of androgens and reduce the production of sex hormone-binding globulin in the liver. This leads to increased levels of free circulating androgens, which inhibit lipolysis and promote lipogenesis, resulting in insulin resistance, visceral fat accumulation, and endothelial dysfunction (44). Meanwhile, women with long or irregular menstrual cycles have metabolic dysfunctions such as higher incidence rate of diabetes and hypertension, higher BMI, higher total cholesterol levels, and lower HDL-C levels (5), which may increase the risk of CVD. Polycystic ovary syndrome, characterized by obesity, hyperandrogenism, and insulin resistance, accounting for the most cases of cycle irregularities or oligomenorrhea, is associated with cardiovascular risks (45) by mediating impaired endothelium dependent vasodilation, inflammatory activation, coronary artery calcification, and dyslipidemia (44, 46). Nevertheless, previous mendelian randomization studies did not found association of genetically predicted polycystic ovary syndrome with risk of coronary heart disease (47, 48), indicating that our results are not affected by confounding role of polycystic ovary syndrome. Other mechanisms yet to be fully elucidated are also suspected to play a role. The luteinizing hormone (LH) surge induces the release of numerous inflammatory mediators (49), and menstrual disorders may favor pro-inflammatory process, which may contribute to CVD (10). Further studies are required to reveal the mechanism.

Apart from EM and IM, growing evidence shows that chronic pain is an underestimated cardiovascular risk factor, which is related to the increased risk of myocardial infarction, stroke, heart failure and cardiovascular death (12, 50). A meta-analysis involving 25 large observational studies, including patients with various chronic pain syndromes, found a significant correlation between chronic pain and CVD (51). Previous clinical study showed that primary dysmenorrhea, a common pain in the female reproductive cycle, can be associated with cardiac arrhythmias compared to the control group, especially AF, by increasing P wave dispersion (52). Nevertheless, the relationship between dysmenorrhea and the risk of CVD, has not been elucidated so far. Our MR analysis showed that genetically determined dysmenorrhea is significantly associated with higher risk of AF and IS (cardioembolic). Further multiple sensitivity analyses suggested no evidence of overall pleiotropy, nor heterogeneity between individual genetic instrument estimation, indicating the robustness of the result. Autonomic nervous system including cardiac sympathetic nervous system and parasympathetic nervous system nerves, plays an indispensable role in the modulation of cardiac electrophysiology and arrhythmogenesis (53). The overactivation of sympathetic nervous system in chronic pain may responsible for the above findings (50, 54). Further studies should be done to clarify the underlying pathophysiological mechanisms. Our study highlights the importance of EM, IM, and dysmenorrhea on CVD from a genetic perspective, which holds substantial implications for public health strategies aimed at CVD prevention in women. This study also lays the groundwork for identifying high-risk CVD cardiovascular female patients. Early clinical intervention of menstrual diseases may be necessary, and better management of menstrual disorders and dysmenorrhea may help reduce the risks for certain CVD.

### 4.1 Study strengths

First, three sets of genetic instruments to symbolize menstruation from different aspects (objective menstrual cycle and subjective symptoms) used in this study broaden the scope and generalizability of significant estimates. Notably, for AF, similar results were generated in all sets, which support the robustness of our findings. Second, our MR analysis, along with previous observational studies, highlight the importance of female menstrual health in CVD. Furthermore, investigating dysmenorrhea could shed light on the potential impact of chronic pain on CVD, expanding upon existing evidence (55, 56).

### 4.2 Study limitations

Our study has several limitations. Firstly, the European dataset used does not represent the global population, thus potentially limiting the generalizability of our findings to other populations. Secondly, due to our reliance on participants’ self-reporting of menstrual irregularity, there might be potential misclassifications regarding exposure. Nevertheless, given the large sample size at the summary level, minor misclassifications are unlikely to significantly alter our overall findings. Thirdly, the definition of dysmenorrhea is based on ICD-10-N94 “pain and other conditions associated with female genital organs and menstrual cycle,” and includes non-dysmenorrhea conditions such as dyspareunia and mittelschmerz. However, non-dysmenorrhea cases only account for a small proportion in this data. Fourthly, other factors, such as the use of oral painkillers, might mediate the impact of exposures on outcomes. However, a comprehensive cohort study has indicated that chronic pain is linked to an elevated risk of CVD independent of known cardiovascular risk factors, socioeconomic status, comorbidities, and medication use (12). Therefore, the absence of multivariate analysis may have limited impact on our outcome. In future study, we will increase the sample size and analyze the mediating effects of multiple potential factors. Of note, MR analysis can only explain partial genetic effects. Therefore, the findings should be interpreted with caution, and further studies are needed to confirm the causal effects.

## 5 Conclusion

Evidence from our study supports a causal relationship of excessive menstruation, irregular menses, and dysmenorrhea on cardiovascular outcomes, particularly the association of irregular menses with myocardial infarction. These results were independent of sex hormone levels. These characteristics may serve as markers for identifying women at higher risk of developing CVD in the future. Given that excessive menstruation, irregular menses and dysmenorrhea are among the most common gynecological issues faced by women, our findings, taken together with previous observations, underscore the importance of early clinical intervention of menstrual diseases.

## Data availability statement

The original contributions presented in the study are included in the article/**Supplementary Material**. Further inquiries can be directed to the corresponding authors.

## Ethics statement

Ethical approval was not required for the study involving humans in accordance with the local legislation and institutional requirements. Written informed consent to participate in this study was not required from the participants or the participants’ legal guardians/next of kin in accordance with the national legislation and the institutional requirements.

## Author contributions

SL: Conceptualization, Data curation, Methodology, Writing – original draft, Writing – review & editing, Formal analysis, Visualization. QJ: Writing – review & editing, Data curation, Methodology, Supervision. DW: Methodology, Writing – review & editing, Funding acquisition, Software, Supervision. TL: Conceptualization, Data curation, Writing – review & editing. XW: Funding acquisition, Supervision, Writing – review & editing.
