# Supplementary material for: Effects of menstrual disorders and dysmenorrhea on cardiovascular disease: a Mendelian randomization study
Source: Front Endocrinol (Lausanne). 2024 Feb 5;15:1302312. doi: 10.3389/fendo.2024.1302312 (PMC10875084; doi:10.3389/fendo.2024.1302312)
Supplement: Supplementary file 1 [file DataSheet_1.docx]

Supplementary Material

**Supplementary Figures**


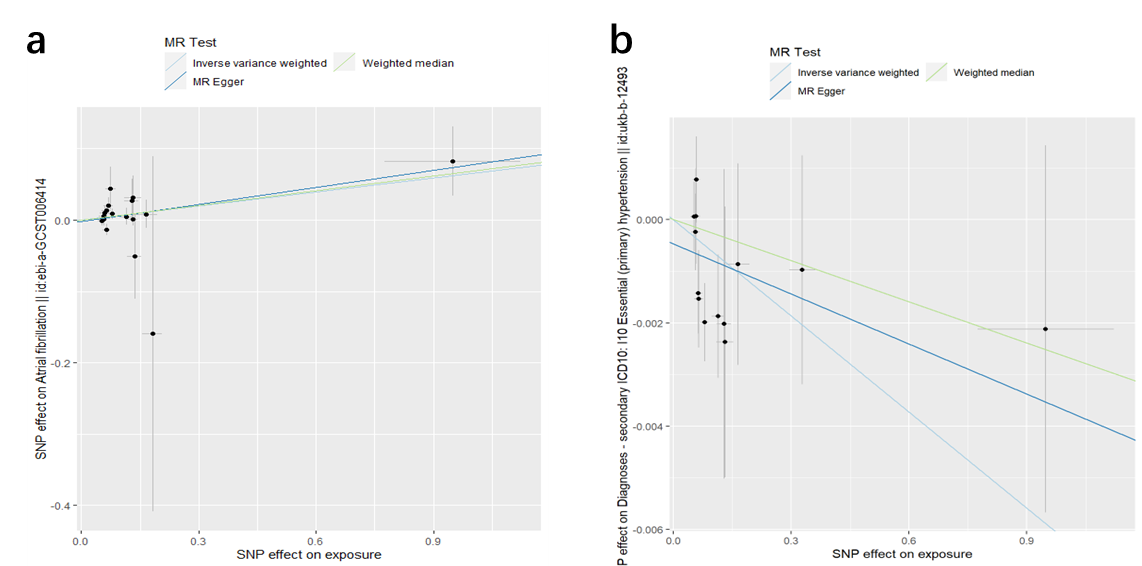


**Supplementary Figure 1.** Scatter plots of nominal significant estimates from genetically predicted excessive menstruation on CVD. (a) genetically predicted EM on AF; (b) genetically predicted EM on HT.


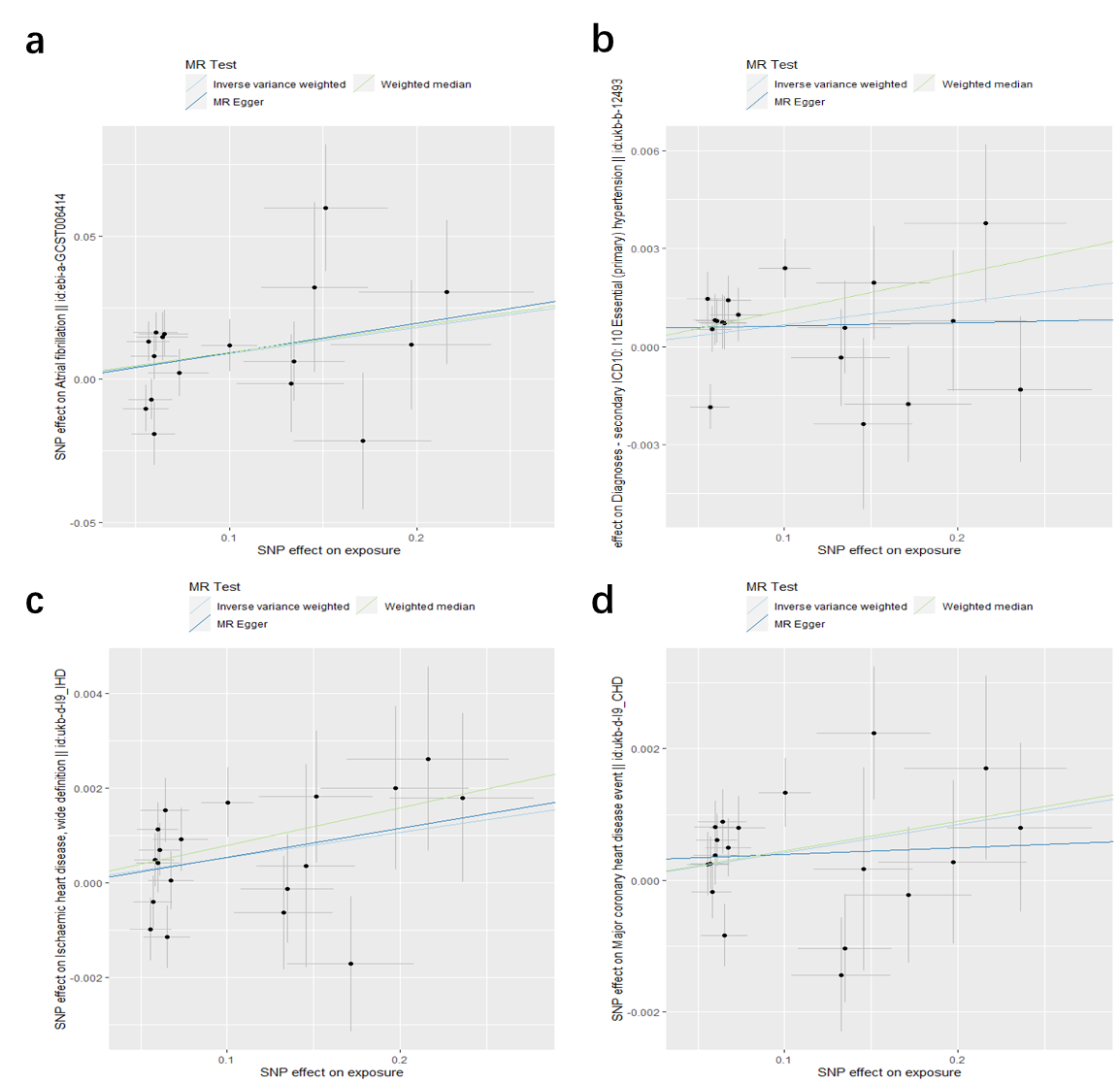


**Supplementary Figure 2.** Scatter plots of nominal significant estimates from genetically predicted IM on CVD. (a) genetically predicted IM on AF; (b) genetically predicted IM on HT; (c) genetically predicted IM on IHD; (d) genetically predicted IM on CHD.


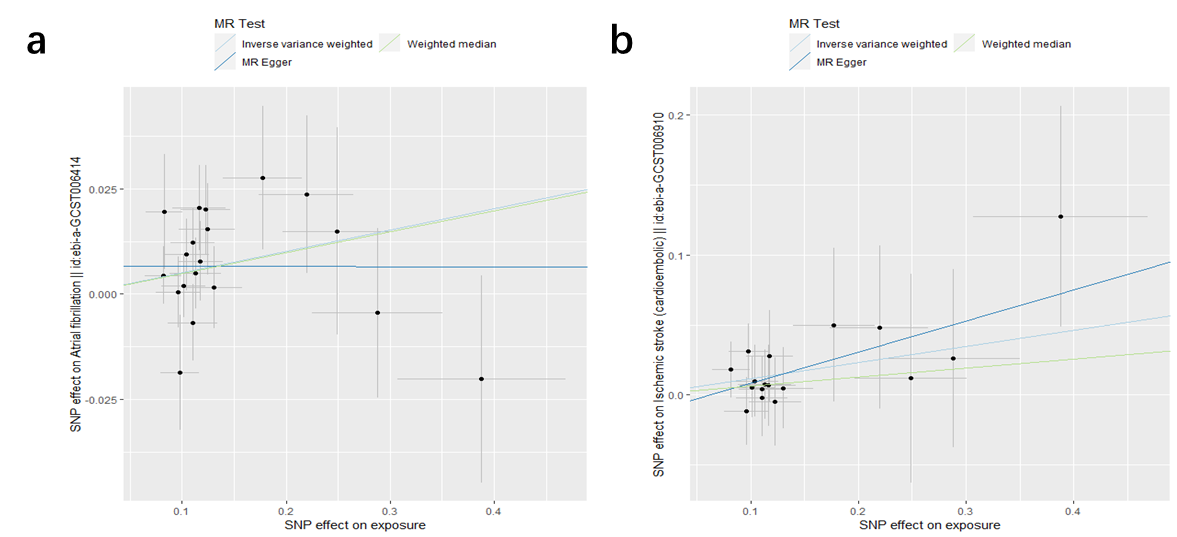


**Supplementary Figure 3.** Scatter plots of nominal significant estimates from genetically predicted dysmenorrhea on CVD. (a) genetically predicted dysmenorrhea on AF; (b) genetically predicted dysmenorrhea on IS.


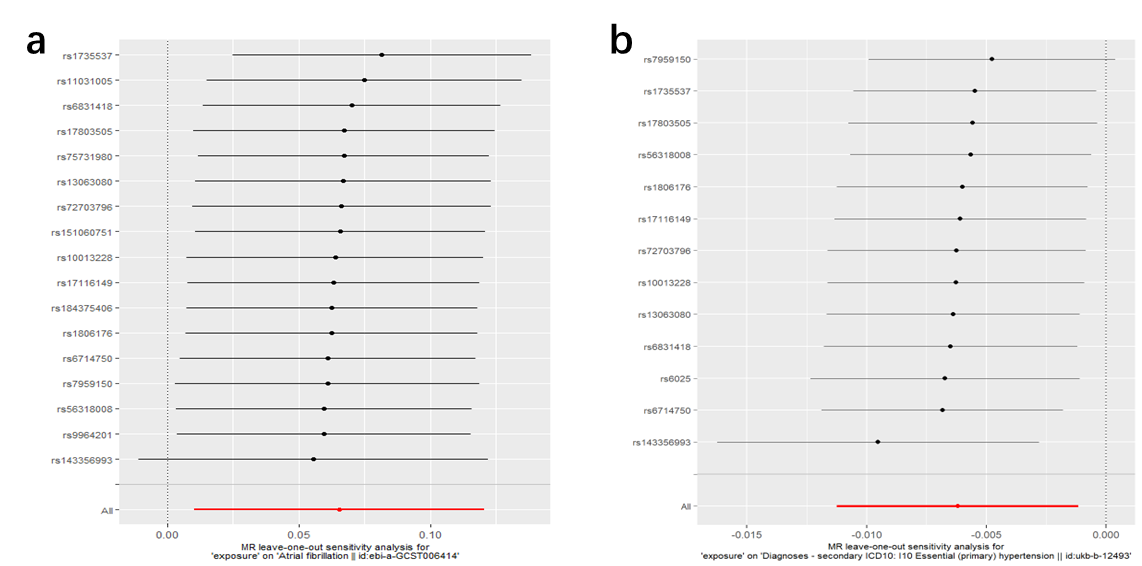


**Supplementary Figure 4.** Leave-one-out plots of nominal significant estimates from genetically predicted EM on CVD. (a) genetically predicted EM on AF; (b) genetically predicted EM on HT.


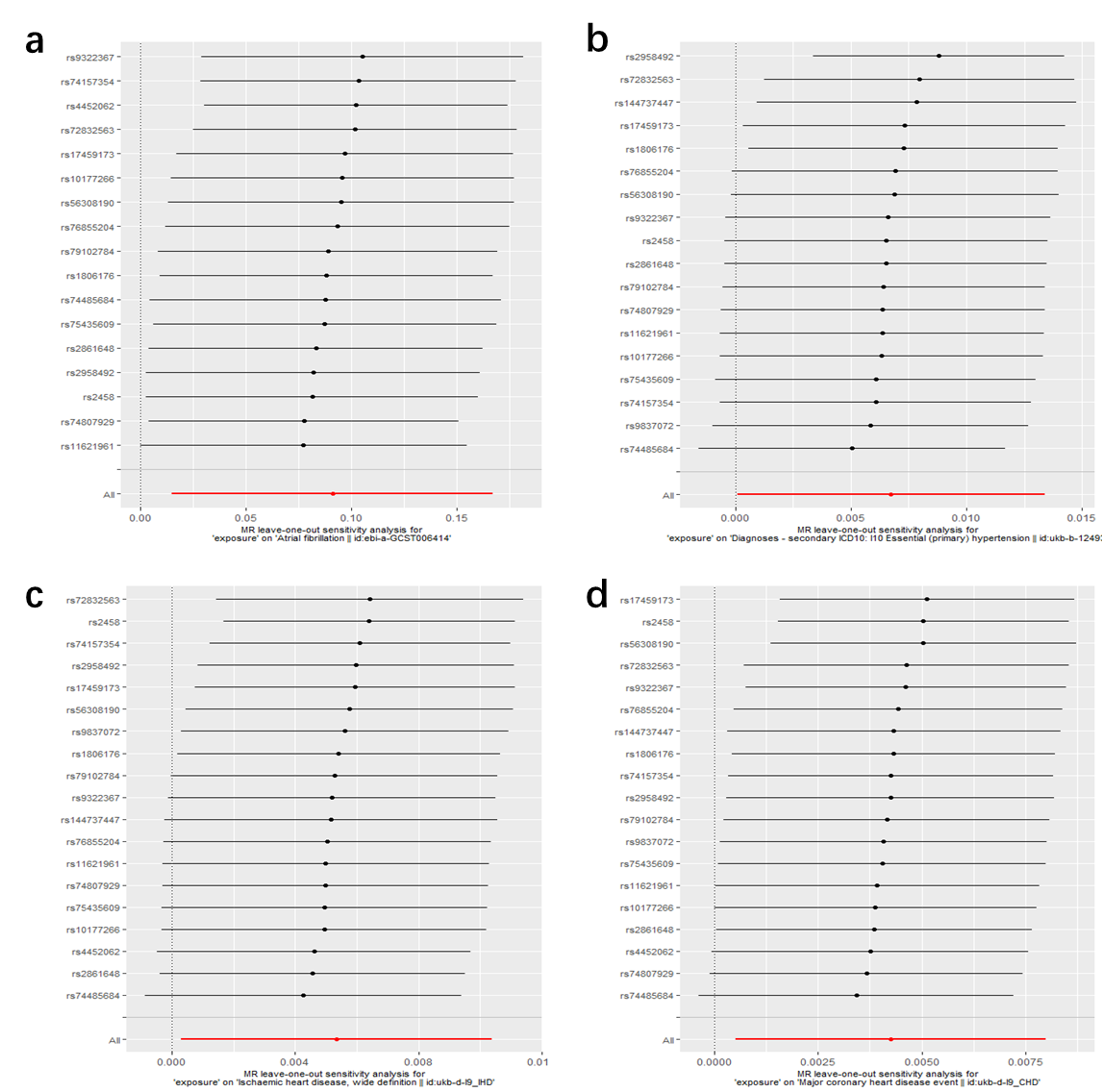


**Supplementary Figure 5.** Leave-one-out plots of nominal significant estimates from genetically predicted IM on CVD. (a) genetically predicted IM on AF; (b) genetically predicted IM on HT; (c) genetically predicted IM on IHD; (d) genetically predicted IM on CHD.


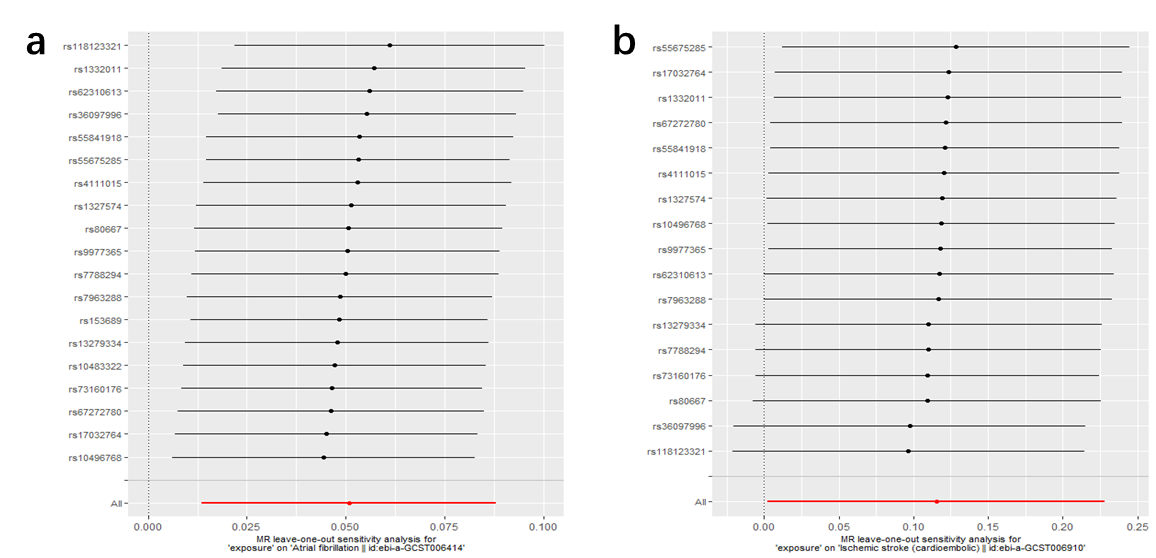


**Supplementary Figure 6.** Leave-one-out plots of nominal significant estimates from genetically predicted dysmenorrhea on CVD. (a) genetically predicted dysmenorrhea on AF; (b) genetically predicted dysmenorrhea on IS.


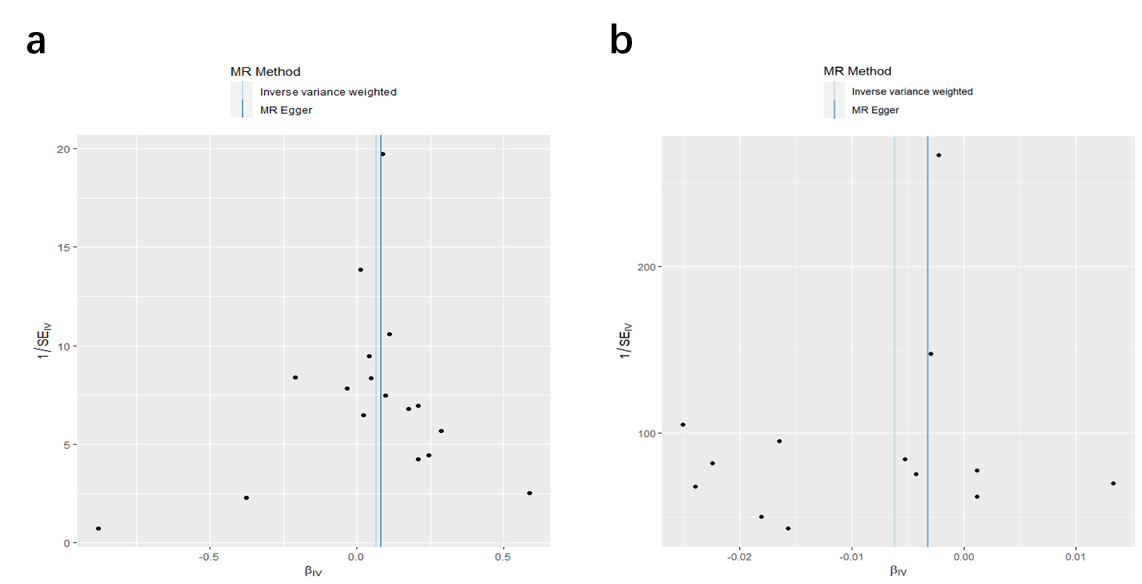


**Supplementary Figure 7.** Funnel plots of nominal significant estimates from genetically predicted EM on CVD. (a) genetically predicted EM on AF; (b) genetically predicted EM on HT.
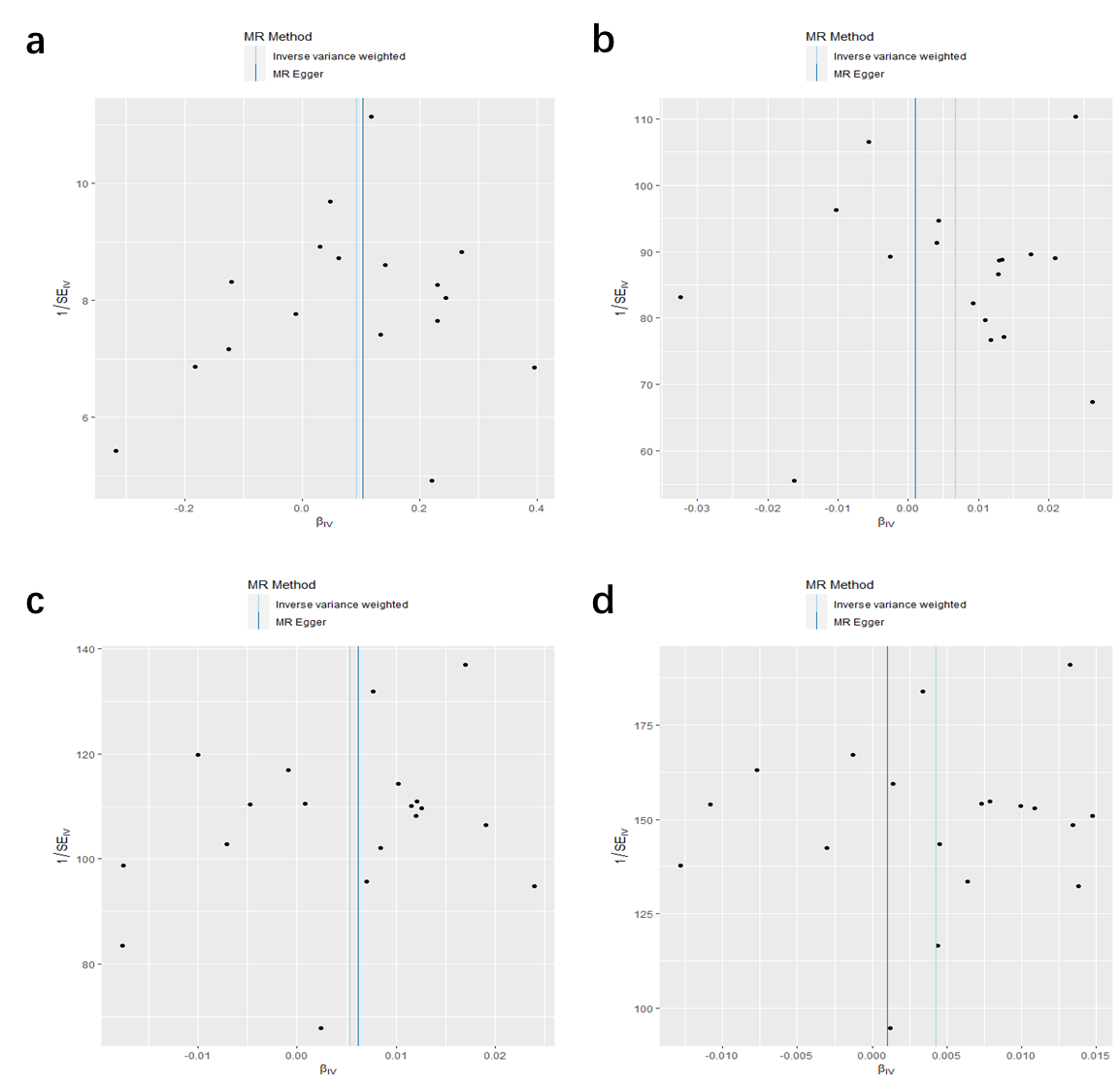


**Supplementary Figure 8.** Funnel plots of nominal significant estimates from genetically predicted IM on CVD. (a) genetically predicted IM on AF; (b) genetically predicted IM on HT; (c) genetically predicted IM on IHD; (d) genetically predicted IM on CHD.


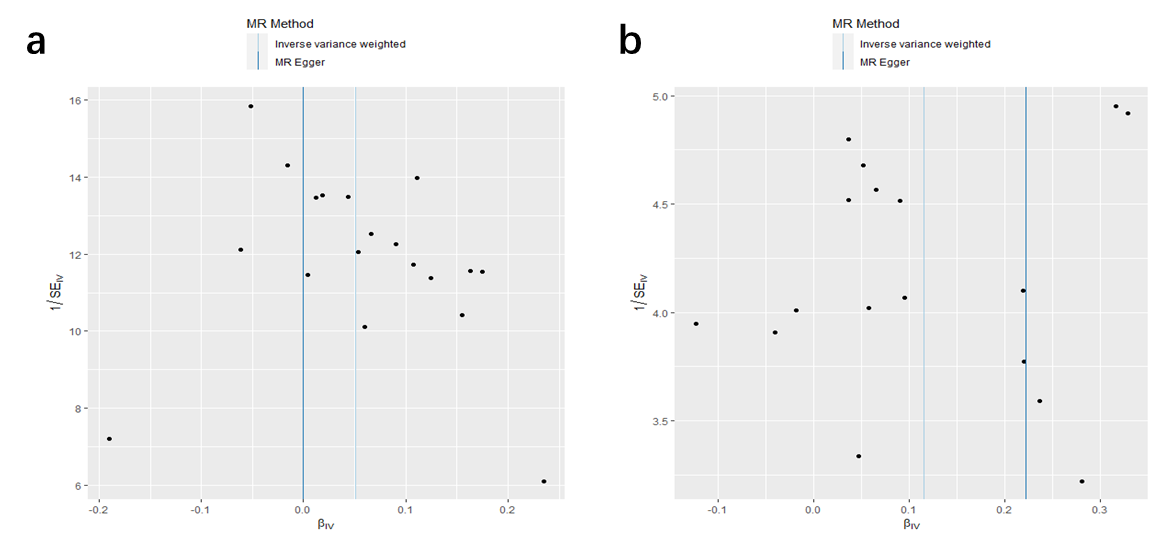


**Supplementary Figure 9.** Funnel plots of nominal significant estimates from genetically predicted dysmenorrhea on CVD. (a) genetically predicted dysmenorrhea on AF; (b) genetically predicted dysmenorrhea on IS.

Abbreviation: EM, excessive menstruation; IM, irregular menses; CVD, cardiovascular disease; AF, atrial fibrillation; HT, hypertension; MI, myocardial infarction; IHD, ischemic heart disease; CHD, coronary heart disease; IS, ischemic stroke (cardioembolic).

# Supplementary Tables

## Table S1. Characteristics of the genetic IVs used in TSMR analysis between menstruation and cardiovascular disease.

| **Phenotype** | **rsID** | **other_allele** | **effect_allele** | **EAF** | **P-value** | **beta** | **SE(beta)** | **N** | **R2** | **F value** |
| --- | --- | --- | --- | --- | --- | --- | --- | --- | --- | --- |
| Excessive menstruation | rs56318008 | C | T | 0.164698 | 4.32793E-08 | 0.0640419 | 0.011693 | 144388 | 0.000208 | 29.996494 |
| Excessive menstruation | rs72703796 | A | G | 0.0238989 | 1.91408E-08 | -0.164582 | 0.029287 | 144388 | 0.000219 | 31.579333 |
| Excessive menstruation | rs6025 | C | T | 0.0199152 | 1.93776E-23 | -0.327527 | 0.032831 | 144388 | 0.000689 | 99.522808 |
| Excessive menstruation | rs6714750 | A | G | 0.289534 | 2.16656E-09 | -0.0583669 | 0.009753 | 144388 | 0.000248 | 35.817284 |
| Excessive menstruation | rs1735537 | T | C | 0.24498 | 3.37054E-10 | 0.0636596 | 0.010136 | 144388 | 0.000273 | 39.446251 |
| Excessive menstruation | rs13063080 | C | T | 0.251891 | 1.3755E-08 | -0.057474 | 0.010125 | 144388 | 0.000223 | 32.220939 |
| Excessive menstruation | rs10013228 | A | G | 0.243376 | 3.92202E-08 | -0.0560929 | 0.010209 | 144388 | 0.000209 | 30.187444 |
| Excessive menstruation | rs6831418 | T | C | 0.529833 | 2.06657E-09 | -0.0523926 | 0.008743 | 144388 | 0.000249 | 35.909385 |
| Excessive menstruation | rs184375406 | G | A | 0.120656 | 3.30986E-08 | -0.075218 | 0.013616 | 144388 | 0.000211 | 30.516315 |
| Excessive menstruation | rs4869733 | G | C | 0.088162 | 5.83848E-12 | 0.104957 | 0.015248 | 144388 | 0.000328 | 47.382047 |
| Excessive menstruation | rs17803505 | T | G | 0.0780144 | 8.44501E-12 | -0.113821 | 0.016663 | 144388 | 0.000323 | 46.658674 |
| Excessive menstruation | rs151060751 | G | A | 0.0301606 | 3.23445E-13 | 0.181658 | 0.024938 | 144388 | 0.000367 | 53.059832 |
| Excessive menstruation | rs75731980 | C | T | 0.0642107 | 1.27174E-14 | 0.136227 | 0.017672 | 144388 | 0.000411 | 59.422905 |
| Excessive menstruation | rs17116149 | G | A | 0.0585118 | 2.61638E-12 | 0.128657 | 0.018388 | 144388 | 0.000339 | 48.956542 |
| Excessive menstruation | rs11031005 | T | C | 0.16753 | 1.1079E-28 | -0.13165 | 0.011849 | 144388 | 0.000854 | 123.45508 |
| Excessive menstruation | rs1806176 | T | C | 0.0439505 | 2.09291E-09 | -0.131126 | 0.021889 | 144388 | 0.000248 | 35.88458 |
| Excessive menstruation | rs7959150 | A | G | 0.738355 | 1.38739E-15 | -0.0793329 | 0.009933 | 144388 | 0.000442 | 63.784391 |
| Excessive menstruation | rs143356993 | T | C | 0.000639033 | 4.87798E-08 | 0.948452 | 0.173846 | 144388 | 0.000206 | 29.764274 |
| Excessive menstruation | rs9964201 | C | A | 0.144126 | 4.42059E-08 | -0.0687707 | 0.012565 | 144388 | 0.000207 | 29.954976 |
| Irregular menses | rs3138157 | T | A | 0.0592843 | 4.88101E-06 | -0.115521 | 0.025279 | 196550 | 0.000106 | 20.883065 |
| Irregular menses | rs144737447 | C | T | 0.0209481 | 1.93072E-08 | -0.235877 | 0.041985 | 196550 | 0.000161 | 31.562712 |
| Irregular menses | rs74807929 | A | G | 0.0332231 | 4.43701E-06 | -0.151556 | 0.03302 | 196550 | 0.000107 | 21.06589 |
| Irregular menses | rs16988102 | C | G | 0.160286 | 2.1425E-06 | -0.0741323 | 0.015641 | 196550 | 0.000114 | 22.462562 |
| Irregular menses | rs2861648 | C | T | 0.793743 | 3.66708E-06 | -0.0642845 | 0.013886 | 196550 | 0.000109 | 21.431252 |
| Irregular menses | rs10177266 | T | G | 0.155664 | 4.50329E-06 | -0.073149 | 0.015948 | 196550 | 0.000107 | 21.037516 |
| Irregular menses | rs73000137 | C | G | 0.163743 | 4.82214E-06 | -0.0712796 | 0.015589 | 196550 | 0.000106 | 20.90636 |
| Irregular menses | rs79102784 | T | G | 0.277121 | 2.5567E-06 | -0.0601064 | 0.012779 | 196550 | 0.000113 | 22.123308 |
| Irregular menses | rs9837072 | A | C | 0.756788 | 4.40413E-07 | 0.067624 | 0.013389 | 196550 | 0.00013 | 25.508302 |
| Irregular menses | rs75435609 | G | A | 0.0165948 | 3.77563E-06 | -0.216062 | 0.046733 | 196550 | 0.000109 | 21.375221 |
| Irregular menses | rs11742908 | C | G | 0.190143 | 4.25177E-06 | 0.0664931 | 0.014459 | 196550 | 0.000108 | 21.147557 |
| Irregular menses | rs17459173 | A | C | 0.0460808 | 3.31826E-06 | -0.132778 | 0.028554 | 196550 | 0.00011 | 21.622747 |
| Irregular menses | rs9322367 | T | C | 0.604136 | 4.96089E-07 | -0.0582065 | 0.011577 | 196550 | 0.000129 | 25.278656 |
| Irregular menses | rs56308190 | T | C | 0.0497228 | 6.44629E-07 | -0.134726 | 0.027068 | 196550 | 0.000126 | 24.773609 |
| Irregular menses | rs76855204 | A | C | 0.0200376 | 3.92121E-06 | -0.197067 | 0.042697 | 196550 | 0.000108 | 21.302806 |
| Irregular menses | rs2458 | G | A | 0.232697 | 9.45214E-07 | 0.0651774 | 0.013294 | 196550 | 0.000122 | 24.036529 |
| Irregular menses | rs2958492 | T | C | 0.554123 | 5.9484E-07 | -0.0570564 | 0.011428 | 196550 | 0.000127 | 24.928391 |
| Irregular menses | rs74157354 | G | A | 0.334857 | 4.1709E-06 | -0.0556753 | 0.012096 | 196550 | 0.000108 | 21.184373 |
| Irregular menses | rs74485684 | T | C | 0.181586 | 2.13059E-11 | -0.10024 | 0.014968 | 196550 | 0.000228 | 44.846932 |
| Irregular menses | rs1806176 | T | C | 0.0441161 | 3.60662E-07 | -0.145643 | 0.028621 | 196550 | 0.000132 | 25.894 |
| Irregular menses | rs76171884 | G | C | 0.034151 | 2.74138E-06 | -0.152812 | 0.032587 | 196550 | 0.000112 | 21.989418 |
| Irregular menses | rs11621961 | C | T | 0.350243 | 4.0205E-07 | -0.0609417 | 0.012025 | 196550 | 0.000131 | 25.684374 |
| Irregular menses | rs72832563 | C | T | 0.0268218 | 3.16155E-06 | -0.171474 | 0.036797 | 196550 | 0.00011 | 21.715728 |
| Irregular menses | rs4452062 | T | C | 0.623978 | 3.18346E-07 | -0.0598008 | 0.011698 | 196550 | 0.000133 | 26.135058 |
| Dysmenorrhea | rs17032764 | C | T | 0.147011 | 4.15987E-07 | 0.122633 | 0.024229 | 114540 | 0.000224 | 25.618099 |
| Dysmenorrhea | rs36097996 | T | C | 0.357711 | 1.11004E-07 | -0.098027 | 0.018469 | 114540 | 0.000246 | 28.171274 |
| Dysmenorrhea | rs55841918 | C | T | 0.107663 | 2.65131E-06 | 0.130534 | 0.027796 | 114540 | 0.000193 | 22.053218 |
| Dysmenorrhea | rs10496768 | G | A | 0.145476 | 4.80828E-06 | -0.116548 | 0.025486 | 114540 | 0.000183 | 20.911987 |
| Dysmenorrhea | rs3197999 | G | A | 0.393883 | 3.06817E-07 | -0.0930946 | 0.018185 | 114540 | 0.000229 | 26.205983 |
| Dysmenorrhea | rs9288996 | C | G | 0.247417 | 1.79875E-09 | 0.120564 | 0.020044 | 114540 | 0.000316 | 36.179558 |
| Dysmenorrhea | rs62310613 | C | T | 0.0239734 | 4.14744E-06 | -0.287677 | 0.062486 | 114540 | 0.000185 | 21.195051 |
| Dysmenorrhea | rs270215 | G | C | 0.510077 | 1.86763E-06 | 0.0854641 | 0.017927 | 114540 | 0.000198 | 22.726117 |
| Dysmenorrhea | rs153689 | T | C | 0.530072 | 2.90944E-06 | -0.0830887 | 0.017765 | 114540 | 0.000191 | 21.874892 |
| Dysmenorrhea | rs55675285 | C | T | 0.238644 | 4.36204E-06 | -0.0962748 | 0.02096 | 114540 | 0.000184 | 21.098304 |
| Dysmenorrhea | rs17679286 | A | G | 0.0492501 | 3.20915E-06 | -0.19938 | 0.042813 | 114540 | 0.000189 | 21.686936 |
| Dysmenorrhea | rs1332011 | T | C | 0.173417 | 3.71099E-06 | -0.110301 | 0.023839 | 114540 | 0.000187 | 21.408138 |
| Dysmenorrhea | rs7788294 | C | T | 0.802079 | 6.39396E-08 | -0.117743 | 0.021774 | 114540 | 0.000255 | 29.239519 |
| Dysmenorrhea | rs13279334 | C | T | 0.0449819 | 1.51607E-06 | -0.219478 | 0.045638 | 114540 | 0.000202 | 23.127 |
| Dysmenorrhea | rs66531120 | A | C | 0.199239 | 2.28676E-06 | -0.106205 | 0.022471 | 114540 | 0.000195 | 22.337256 |
| Dysmenorrhea | rs10217463 | C | G | 0.0517444 | 4.8791E-06 | 0.173883 | 0.038049 | 114540 | 0.000182 | 20.883873 |
| Dysmenorrhea | rs1327574 | G | A | 0.139472 | 4.87742E-06 | 0.113206 | 0.024772 | 114540 | 0.000182 | 20.884288 |
| Dysmenorrhea | rs4111015 | C | T | 0.769456 | 1.61317E-06 | 0.101515 | 0.021164 | 114540 | 0.000201 | 23.00773 |
| Dysmenorrhea | rs118123321 | C | T | 0.0150871 | 1.73848E-06 | -0.387991 | 0.081141 | 114540 | 0.0002 | 22.863922 |
| Dysmenorrhea | rs7963288 | T | C | 0.808097 | 2.12378E-06 | -0.10413 | 0.021963 | 114540 | 0.000196 | 22.479187 |
| Dysmenorrhea | rs73160176 | G | A | 0.051489 | 3.12903E-06 | 0.177167 | 0.038001 | 114540 | 0.00019 | 21.73542 |
| Dysmenorrhea | rs10483322 | G | A | 0.126931 | 4.72128E-06 | -0.12402 | 0.027098 | 114540 | 0.000183 | 20.946787 |
| Dysmenorrhea | rs67272780 | C | A | 0.215703 | 1.40945E-07 | 0.110352 | 0.020964 | 114540 | 0.000242 | 27.70928 |
| Dysmenorrhea | rs9977365 | C | T | 0.0329583 | 1.93508E-06 | -0.248771 | 0.052262 | 114540 | 0.000198 | 22.657998 |
| Dysmenorrhea | rs80667 | T | C | 0.513787 | 3.42752E-06 | -0.0819766 | 0.017655 | 114540 | 0.000188 | 21.560626 |
| Abbreviation: EAF, effect allele frequency; SE, standard error. | | | | | | | | | | |

## Table S2. Characteristics of each step in TSMR analysis.

| **exposure** | **outcome** | **Nsnps** | **P-value** | **LD** | **outcome lack of  proxies** | **Palindromic and  incompatible SNPs** | **horizontal pleiotropy** | **outliers** |
| --- | --- | --- | --- | --- | --- | --- | --- | --- |
| Excessive  menstruation | Atrial fibrillation | 16 | 1110 （P<5E-8） | 1091 | 1 | rs4869733 | rs11031005: Sex hormone levels; Ever used hormone-replacement therapy | NA |
|  | hypertension | 13 |  |  | 3 |  |  | rs9964201 |
|  | Myocardial infarction | 15 |  |  | 2 |  |  | NA |
|  | Ischaemic heart disease | 15 |  |  | 2 |  |  | NA |
|  | Coronary heart disease | 15 |  |  | 2 |  |  | NA |
|  | Ischemic stroke | 13 |  |  | 4 |  |  | NA |
| Irregular  menses | Atrial fibrillation | 17 | 229 （P<5E-6） | 205 | 1 | rs11742908 rs16988102 rs3138157 rs73000137 rs76171884 | NA | rs144737447 |
|  | hypertension | 18 |  |  | 1 |  | NA | NA |
|  | Myocardial infarction | 18 |  |  | 0 |  | NA | rs2458 |
|  | Ischaemic heart disease | 19 |  |  | 0 |  | NA | NA |
|  | Coronary heart disease | 19 |  |  | 0 |  | NA | NA |
|  | Ischemic stroke | 18 |  |  | 1 |  | NA | NA |
| Dysmenorrhea | Atrial fibrillation | 19 | 376 （P<5E-6） | 351 | 0 | rs10217463 rs270215 rs9288996 | rs17679286: CAD rs3197999: DBP、CAD rs66531120: Vascular or heart problems diagnosed by doctor: high blood pressure | NA |
|  | hypertension | 17 |  |  | 1 |  |  | rs10496768 |
|  | Myocardial infarction | 19 |  |  | 0 |  |  | NA |
|  | Ischaemic heart disease | 19 |  |  | 0 |  |  | NA |
|  | Coronary heart disease | 19 |  |  | 0 |  |  | NA |
|  | Ischemic stroke | 17 |  |  | 2 |  |  | NA |
| LD: Linkage disequilibrium (r2 < 0.001, distance 1Mb); horizontal pleiotropy: detected by PhenoScanner; outliers: detected by MR-PRESSO.  Abbreviation: CAD, coronary artery disease; DBP, diastolic blood pressure; Nsnps: number of single nucleotide polymorphisms. | | | | | | | | |

## Table S3. Mendelian randomization analysis between menstruation and cardiovascular diseases.

| **Exposure** | **Outcome** | **N_IVs** | **MR method** | **beta** | **SE** | **P value** | **lo_ci** | **up_ci** | **OR** | **or_lci95** | **or_uci95** |
| --- | --- | --- | --- | --- | --- | --- | --- | --- | --- | --- | --- |
| Excessive menstruation | Atrial fibrillation | 16 | MR Egger | 0.091924 | 0.050243 | 0.088687 | -0.00655285 | 0.190401 | 1.096282 | 0.993469 | 1.209735 |
|  |  |  | WM | 0.086327 | 0.044742 | 0.053675 | -0.00136694 | 0.17402 | 1.090162 | 0.998634 | 1.19008 |
|  |  |  | IVW | 0.075021 | 0.030629 | 0.014312 | 0.014987954 | 0.135054 | 1.077907 | 1.015101 | 1.144599 |
|  | hypertension | 13 | MR Egger | -0.00323 | 0.003535 | 0.380417 | -0.0101576 | 0.003698 | 0.996775 | 0.989894 | 1.003705 |
|  |  |  | WM | -0.00266 | 0.003561 | 0.455639 | -0.00963746 | 0.004323 | 0.997347 | 0.990409 | 1.004333 |
|  |  |  | IVW | -0.0062 | 0.002572 | 0.015853 | -0.01124597 | -0.00116 | 0.993815 | 0.988817 | 0.998837 |
|  | Myocardial infarction | 15 | MR Egger | -0.0155 | 0.068674 | 0.824945 | -0.15010112 | 0.119102 | 0.98462 | 0.860621 | 1.126485 |
|  |  |  | WM | 0.020344 | 0.056716 | 0.719822 | -0.09081908 | 0.131507 | 1.020552 | 0.913183 | 1.140545 |
|  |  |  | IVW | 0.036282 | 0.048267 | 0.452242 | -0.05832215 | 0.130886 | 1.036948 | 0.943346 | 1.139837 |
|  | Ischaemic heart disease | 15 | MR Egger | -0.0001 | 0.003546 | 0.977724 | -0.00705082 | 0.006849 | 0.999899 | 0.992974 | 1.006872 |
|  |  |  | WM | 0.000951 | 0.002797 | 0.733907 | -0.00453201 | 0.006434 | 1.000951 | 0.995478 | 1.006455 |
|  |  |  | IVW | 0.002318 | 0.002497 | 0.353243 | -0.00257631 | 0.007213 | 1.002321 | 0.997427 | 1.007239 |
|  | Coronary heart disease | 15 | MR Egger | 0.000269 | 0.002102 | 0.900241 | -0.00385091 | 0.004388 | 1.000269 | 0.996157 | 1.004398 |
|  |  |  | WM | 6.58E-05 | 0.001988 | 0.97358 | -0.00383056 | 0.003962 | 1.000066 | 0.996177 | 1.00397 |
|  |  |  | IVW | -5.6E-05 | 0.001438 | 0.968908 | -0.00287551 | 0.002763 | 0.999944 | 0.997129 | 1.002767 |
|  | Ischemic stroke | 13 | MR Egger | 0.130657 | 0.486057 | 0.79305 | -0.8220135 | 1.083328 | 1.139577 | 0.439546 | 2.954496 |
|  |  |  | WM | -0.09445 | 0.167555 | 0.572947 | -0.4228612 | 0.233954 | 0.90987 | 0.65517 | 1.263587 |
|  |  |  | IVW | -0.06916 | 0.13687 | 0.613327 | -0.3374303 | 0.199101 | 0.933173 | 0.713602 | 1.220305 |
| Irregular menses | Atrial fibrillation | 17 | MR Egger | 0.102985 | 0.104651 | 0.340685 | -0.10213098 | 0.3081 | 1.108474 | 0.902911 | 1.360838 |
|  |  |  | WM | 0.093251 | 0.045202 | 0.039113 | 0.004655484 | 0.181846 | 1.097737 | 1.004666 | 1.19943 |
|  |  |  | IVW | 0.090993 | 0.038972 | 0.019552 | 0.014607799 | 0.167379 | 1.095262 | 1.014715 | 1.182202 |
|  | hypertension | 18 | MR Egger | 0.000992 | 0.008122 | 0.904322 | -1.49E-02 | 0.016912 | 1.000992 | 0.985183 | 1.017056 |
|  |  |  | WM | 0.011108 | 0.00401 | 0.005602 | 3.25E-03 | 0.018968 | 1.01117 | 1.003254 | 1.019149 |
|  |  |  | IVW | 0.006731 | 0.003394 | 0.047323 | 7.94E-05 | 0.013382 | 1.006753 | 1.000079 | 1.013472 |
|  | Myocardial infarction | 18 | MR Egger | 0.198421 | 0.123694 | 0.12824 | -0.04401918 | 0.44086 | 1.219475 | 0.956936 | 1.554044 |
|  |  |  | WM | 0.143298 | 0.064365 | 0.025993 | 0.01714194 | 0.269454 | 1.154074 | 1.01729 | 1.309249 |
|  |  |  | IVW | 0.158484 | 0.050978 | 0.001878 | 0.05856728 | 0.2584 | 1.171733 | 1.060316 | 1.294857 |
|  | Ischaemic heart disease | 19 | MR Egger | 0.006135 | 0.006347 | 0.347237 | -0.00630396 | 0.018575 | 1.006154 | 0.993716 | 1.018748 |
|  |  |  | WM | 0.007937 | 0.003129 | 0.011202 | 0.001803569 | 0.014071 | 1.007969 | 1.001805 | 1.01417 |
|  |  |  | IVW | 0.005351 | 0.002571 | 0.037399 | 0.000312065 | 0.01039 | 1.005365 | 1.000312 | 1.010444 |
|  | Coronary heart disease | 19 | MR Egger | 0.000993 | 0.004638 | 0.832933 | -0.00809674 | 0.010084 | 1.000994 | 0.991936 | 1.010135 |
|  |  |  | WM | 0.00449 | 0.00236 | 0.057121 | -0.00013597 | 0.009116 | 1.0045 | 0.999864 | 1.009157 |
|  |  |  | IVW | 0.004256 | 0.00191 | 0.025916 | 0.000511001 | 0.008 | 1.004265 | 1.000511 | 1.008032 |
|  | Ischemic stroke | 18 | MR Egger | 0.02704 | 0.259156 | 0.918198 | -0.4809056 | 0.534985 | 1.027409 | 0.618223 | 1.707423 |
|  |  |  | WM | 0.009047 | 0.122581 | 0.941164 | -0.2312109 | 0.249306 | 1.009088 | 0.793572 | 1.283134 |
|  |  |  | IVW | -0.0219 | 0.094289 | 0.816344 | -0.2067044 | 0.162907 | 0.97834 | 0.81326 | 1.176928 |
| Dysmenorrhea | Atrial fibrillation | 19 | MR Egger | -0.00046 | 0.05617 | 0.993563 | -0.11055222 | 0.109632 | 0.99954 | 0.89534 | 1.115868 |
|  |  |  | WM | 0.049447 | 0.027237 | 0.069455 | -0.00393721 | 0.102832 | 1.05069 | 0.996071 | 1.108305 |
|  |  |  | IVW | 0.050744 | 0.019029 | 0.007661 | 0.013447348 | 0.088041 | 1.052054 | 1.013538 | 1.092033 |
|  | hypertension | 17 | MR Egger | -0.0032 | 0.007028 | 0.65563 | -0.01697363 | 0.010578 | 0.996807 | 0.98317 | 1.010634 |
|  |  |  | WM | -0.00192 | 0.002866 | 0.502409 | -0.00753908 | 0.003695 | 0.99808 | 0.992489 | 1.003702 |
|  |  |  | IVW | -0.00115 | 0.002426 | 0.635888 | -0.00590467 | 0.003607 | 0.998852 | 0.994113 | 1.003614 |
|  | Myocardial infarction | 19 | MR Egger | -0.07633 | 0.092213 | 0.419293 | -0.25706371 | 0.10441 | 0.926513 | 0.773319 | 1.110055 |
|  |  |  | WM | 0.044345 | 0.040376 | 0.272073 | -0.03479203 | 0.123482 | 1.045343 | 0.965806 | 1.13143 |
|  |  |  | IVW | 0.023392 | 0.032188 | 0.467391 | -0.03969649 | 0.086481 | 1.023668 | 0.961081 | 1.09033 |
|  | Ischaemic heart disease | 19 | MR Egger | -0.00699 | 0.004619 | 0.148792 | -0.01604012 | 0.002067 | 0.993038 | 0.984088 | 1.00207 |
|  |  |  | WM | 0.00022 | 0.002138 | 0.917911 | -0.00397084 | 0.004412 | 1.00022 | 0.996037 | 1.004421 |
|  |  |  | IVW | -0.00024 | 0.001673 | 0.885099 | -0.00352086 | 0.003037 | 0.999758 | 0.996485 | 1.003042 |
|  | Coronary heart disease | 19 | MR Egger | 0.000489 | 0.003023 | 0.873404 | -0.00543612 | 0.006414 | 1.000489 | 0.994579 | 1.006435 |
|  |  |  | WM | -0.00095 | 0.001434 | 0.509031 | -0.00375831 | 0.001864 | 0.999053 | 0.996249 | 1.001866 |
|  |  |  | IVW | 0.000697 | 0.001026 | 0.496931 | -0.00131387 | 0.002708 | 1.000697 | 0.998687 | 1.002711 |
|  | Ischemic stroke | 17 | MR Egger | 0.222412 | 0.176645 | 0.22724 | -0.12381258 | 0.568637 | 1.249086 | 0.883545 | 1.765858 |
|  |  |  | WM | 0.064164 | 0.074759 | 0.390741 | -0.08236401 | 0.210692 | 1.066267 | 0.920937 | 1.234532 |
|  |  |  | IVW | 0.115289 | 0.057712 | 0.045753 | 0.002173914 | 0.228404 | 1.122198 | 1.002176 | 1.256593 |
| Abbreviation: WM, weighted median; IVW, inverse variance weighted; OR, odds ratio; SE, standard error. | | | | | | | | | | | |

## Table S4. Sensitivity analyses of causal relationship of menstrual disorder and dysmenorrhea on cardiovascular disease by Cochran’s Q test.

| Exposure | Outcome | Cochran’s Q | | | |
| --- | --- | --- | --- | --- | --- |
|  |  | MR-egger Q value | MR-egger P value | IVW Q value | IVW P value |
| Excessive menstruation | Atrial fibrillation | 13.62391 | 0.4780889 | 13.80404 | 0.5404405 |
|  | Hypertension | 10.9546 | 0.4470743 | 12.40668 | 0.4135954 |
|  | Myocardial infarction | 18.34136 | 0.1449832 | 19.9141 | 0.1328732 |
|  | Ischaemic heart disease | 20.22986 | 0.08962504 | 21.67423 | 0.08556046 |
|  | Coronary heart disease | 13.83628 | 0.3854742 | 13.88695 | 0.4581672 |
|  | Ischemic stroke | 17.36694 | 0.09748058 | 17.65858 | 0.1264572 |
| Irregular menses | Atrial fibrillation | 26.73836 | 0.03094182 | 26.76582 | 0.04416049 |
|  | Hypertension | 25.8011 | 0.05689192 | 26.78122 | 0.06135541 |
|  | Myocardial infarction | 22.93956 | 0.1153599 | 23.12161 | 0.1453334 |
|  | Ischaemic heart disease | 26.336 | 0.06855266 | 26.36463 | 0.09170838 |
|  | Coronary heart disease | 27.37634 | 0.05277428 | 28.34031 | 0.05706171 |
|  | Ischemic stroke | 20.67917 | 0.191175 | 20.73278 | 0.2384717 |
| Dysmenorrhea | Atrial fibrillation | 16.56504 | 0.4841903 | 17.5038 | 0.4887618 |
|  | Hypertension | 27.074 | 0.02813915 | 27.2496 | 0.03879092 |
|  | Myocardial infarction | 20.84815 | 0.2331565 | 22.47723 | 0.2114847 |
|  | Ischaemic heart disease | 21.51856 | 0.2039495 | 24.59044 | 0.1366197 |
|  | Coronary heart disease | 17.93992 | 0.3926408 | 17.9456 | 0.4592416 |
|  | Ischemic stroke | 4.556401 | 0.9952626 | 4.968103 | 0.9959096 |

## Table S5. Sensitivity analyses of causal relationship of menstrual disorder and dysmenorrhea on cardiovascular disease by MR-Egger and MR-PRESSO.

| Exposure | Outcome | MR-Egger | | | MR-PRESSO | |
| --- | --- | --- | --- | --- | --- | --- |
|  |  | egger_intercept | SE | P value | Global test P-value | Distortion test P-value |
| Excessive menstruation | Atrial fibrillation | -0.00190517 | 0.004488999 | 0.6777173 | 0.578 | NA |
|  | Hypertension | -0.000473573 | 0.000392999 | 0.2534788 | 0.402 | 0.739 |
|  | Myocardial infarction | 0.007806367 | 0.007393747 | 0.3102835 | 0.211 | NA |
|  | Ischaemic heart disease | 0.000373277 | 0.00038745 | 0.3529178 | 0.155 | NA |
|  | Coronary heart disease | -5.01088E-05 | 0.000229664 | 0.8306738 | 0.556 | NA |
|  | Ischemic stroke | -0.01468963 | 0.03417804 | 0.6756414 | 0.129 | NA |
| Irregular menses | Atrial fibrillation | -0.001030876 | 0.008305274 | 0.9028659 | 0.072 | 0.526 |
|  | Hypertension | 0.00053708 | 0.000688904 | 0.4469972 | 0.061 | NA |
|  | Myocardial infarction | -0.003693451 | 0.01036494 | 0.7262407 | 0.161 | 0.658 |
|  | Ischaemic heart disease | -7.14E-05 | 0.000525103 | 0.8934637 | 0.106 | NA |
|  | Coronary heart disease | 0.000296886 | 0.000383725 | 0.4497379 | 0.065 | NA |
|  | Ischemic stroke | -0.004074213 | 0.02000476 | 0.8411853 | 0.226 | NA |
| Dysmenorrhea | Atrial fibrillation | 0.006709755 | 0.006925164 | 0.3461815 | 0.527 | NA |
|  | Hypertension | 0.000274067 | 0.000878688 | 0.7594066 | 0.046 | 0.823 |
|  | Myocardial infarction | 0.01285307 | 0.01115178 | 0.2650418 | 0.242 | NA |
|  | Ischaemic heart disease | 0.000873641 | 0.000560807 | 0.1376945 | 0.154 | NA |
|  | Coronary heart disease | 2.69352E-05 | 0.000367012 | 0.9423519 | 0.489 | NA |
|  | Ischemic stroke | -0.0136302 | 0.02124275 | 0.5307903 | 0.995 | NA |
| Abbreviation: SE, standard error. | | | | | | |
